# A Micro-Level Analysis of Physiological Responses to COVID-19: Continuous Monitoring of Pregnant Women in California

**DOI:** 10.3389/fpubh.2022.808763

**Published:** 2022-04-07

**Authors:** Tamara Jimah, Priscilla Kehoe, Holly Borg, Pamela Pimentel, Amir Rahmani, Nikil Dutt, Yuqing Guo

**Affiliations:** ^1^Sue and Bill Gross School of Nursing, University of California, Irvine, Irvine, CA, United States; ^2^Donald Bren School of Information and Computer Sciences, University of California, Irvine, Irvine, CA, United States; ^3^Institute for Future Health, University of California, Irvine, Irvine, CA, United States

**Keywords:** COVID-19, cardiorespiratory response, sleep, pregnancy, wearable technology

## Abstract

Continuous monitoring of perinatal women in a descriptive case study allowed us the opportunity to examine the time during which the COVID-19 infection led to physiological changes in two low-income pregnant women. An important component of this study was the use of a wearable sensor device, the *Oura* ring, to monitor and record vital physiological parameters during sleep. Two women in their second and third trimesters, respectively, were selected based on a positive COVID-19 diagnosis. Both women were tested using the polymerase chain reaction method to confirm the presence of the virus during which time we were able to collect these physiological data. In both cases, we observed 3–6 days of peak physiological changes in resting heart rate (HR), heart rate variability (HRV), and respiratory rate (RR), as well as sleep surrounding the onset of COVID-19 symptoms. The pregnant woman in her third trimester showed a significant increase in resting HR (*p* = 0.006) and RR (*p* = 0.048), and a significant decrease in HRV (*p* = 0.027) and deep sleep duration (*p* = 0.029). She reported experiencing moderate COVID-19 symptoms and did not require hospitalization. At 38 weeks of gestation, she had a normal delivery and gave birth to a healthy infant. The participant in her second trimester showed similar physiological changes during the 3-day peak period. Importantly, these changes appeared to return to the pre-peak levels. Common symptoms reported by both cases included loss of smell and nasal congestion, with one losing her sense of taste. Results suggest the potential to use the changes in cardiorespiratory responses and sleep for real-time monitoring of health and well-being during pregnancy.

## Introduction

In general, pregnant women are vulnerable to infection due to changes in the cardiorespiratory and immune system, which increases susceptibility to COVID-19, particularly for the overweight and obese (1–4). Previous studies have suggested that the intake of plant-based and Mediterranean diets (e.g., high vegetable and fruit content, low fat and sugar) prior to and during pregnancy reduces excessive gestational weight gain and gestational diabetes and improves immune responses to viral infections (5, 6). Inadvertently, the COVID-19 lockdown has increased the intake of high fat meals and sugary foods and reduced physical activity, all of which raise the risk of greater weight gain and/or developing impaired glucose tolerance and gestational diabetes during pregnancy (5–7). Notably, the majority of pregnant women with severe COVID-19 illness were either overweight or obese prior to pregnancy (8, 9).

Of the infected pregnant women presenting with COVID-19 symptoms, about 80–85% experienced mild-to-moderate illness such as fever, sore throat, cough, loss of taste and smell, headache, fatigue, and shortness of breath (8, 10–13). Approximately 15–20% of infected pregnant women have severe or critical conditions requiring hospitalization, mechanical ventilation, and intensive medical care (1, 8, 10–13). Moreover, pregnant women at increased risk of severe illness from COVID-19 have been described as having the following characteristics: being of African American or Hispanic descent, having a high body-mass index (BMI), and having underlying medical or pregnancy-related health conditions in the second or third trimester (2, 3, 8, 9, 11). Severe COVID-19 conditions during pregnancy increase the odds of adverse perinatal outcomes such as undergoing a cesarean section, experiencing a preterm delivery, or admission of newborns to the neonatal intensive care unit (3, 6, 14).

The availability of mobile health (mHealth) has been instrumental in increasing opportunities to monitor health and promote healthy behaviors, as well as provide treatment to women during pregnancy. For instance, a meta-analysis of 15 randomized controlled trials found mHealth technologies to be effective tools for weight management, controlling gestational diabetes, and promoting mental health (15). In recent years, advanced wearable health technologies, such as smart rings and watches, have provided an opportunity to remotely monitor important physiological measures in patients. For example, studies that used wearable devices to understand the physiological variations surrounding COVID-19 illness in a sample of non-pregnant individuals found elevated levels of resting heart rate, respiratory rate, or total sleep time (16–19). However, there is sparse literature on the continuous monitoring of physiological changes in pregnant women during COVID-19 infection, particularly in terms of the variations in cardiorespiratory responses and sleep. It is important to recognize that the pregnancy period is characterized by distinct cardiorespiratory changes, including increased heart rate and respiratory rate across trimesters (20, 21). In addition, emerging evidence shows that most pregnant women tend to experience changes in sleep patterns (e.g., shorter sleep duration and increased awakenings) and these changes become more pronounced as pregnancy progresses (22–24). It is still unknown how COVID-19 infection impacts these physiological changes during pregnancy. Using a wearable device, i.e., the *Oura* ring, our study aimed to examine variations in the cardiovascular and respiratory systems, as well as changes in sleep of pregnant women diagnosed with COVID-19.

## Methods

### Study Design and Sampling

This was a case study of two pregnant women with a positive COVID-19 diagnosis from a feasibility study that sought to promote physical and mental well-being among perinatal women through mHealth monitoring and recommendations. Study information flyers were distributed on social media and shared with community partners working with low-income pregnant women in Orange County, California. Healthy women with a singleton pregnancy, aged 18–40 years, and with access to a smartphone were eligible to partake in this research. A trained research assistant screened and obtained consent from participants, after which the wearable device (*Oura* ring) was shipped to the consented participants for data collection. Instructions on installing and using the smart ring were provided. The research assistant was also available *via* Zoom to assist with technology set-up. All communication between the study team and participants was conducted virtually according to the US federal stay-at-home order. The selection criterion for this case study was based on a positive COVID-19 diagnosis [confirmed using the polymerase chain reaction (PCR) test] reported by participants during the study period, prior to vaccine availability. Two women met this criterion, with one in her second trimester and another in her third trimester.

### Data Collection Procedure

The study was approved by the university's Institutional Review Board. We trained research assistants to collect self-reported demographic data and COVID-19 symptoms and experiences through virtual visits using the Research Electronic Data Capture (REDCap), a secure web-based survey instrument. Further inquiries were obtained *via* secure text message communication, i.e., OhMD. Objectively, the *Oura* ring was used to collect daily within-subject repeated measures during the night at home to monitor resting heart rate, heart rate variability, respiratory rate, temperature, and sleep. The *Oura* ring (manufactured in Finland) is a titanium band worn around the finger, synchronizes with the participant's mobile phone app that shows various vital physiological indicators during sleep (25). Resting heart rate (HR) is defined as the average heart rate registered, measured by beats per minute (bpm). Resting heart rate variability (HRV), i.e., the variation in time between heartbeats measured in milliseconds (ms), is determined using the root mean square of successive differences of inter-beat interval method (RMSSD). Resting respiratory rate (RR) is measured by the number of breaths taken per minute. Temperature was defined as the skin temperature deviation from the long-term temperature average, measured in Celsius. The duration and stages of sleep were also recorded. Sleep duration included the total number of hours registered from the start to the end of bedtime. Sleep stages comprised the hours in deep, light, and rapid eye movement (REM) sleep.

### Data Analysis

We first examined all data available for the two COVID-19-infected participants in their second and third trimesters (Cases I and II, respectively) for missing data and outliers. Normality was confirmed for all continuous data, followed by a microanalysis of physiological data to capture the specific changes that occurred in the cardiovascular and respiratory system, as well as in the duration and stages of sleep. The means, standard deviations, and paired *t*-tests were calculated for all physiological variables.

Subsequently, standardized Z-scores of the daily changes in selected physiological indicators of interest (i.e., resting heart rate, respiratory rate, and deep sleep) were used for graphical comparison. Statistical significance was set to 0.10 given the case study design (26). The cardiovascular responses to COVID-19 (confirmed by symptom onset and diagnosis) in both participants were sensitive and acutely apparent. This allowed us to use cardiovascular changes to select observational periods (i.e., pre-peak, peak, and post-peak) over the course of the COVID-19 infection. For Case I in her second trimester, the average physiological data including resting HR, HRV, RR, temperature, and the duration and stages of sleep were calculated and compared between the three sets of observation periods surrounding the COVID-19 infection: pre-peak/baseline (4 days prior to the peak cardiovascular response); peak (3 days during the peak cardiovascular response); and post-peak (3 days after the peak cardiovascular response). For Case II in her third trimester, the same average physiological data as described in Case I were available only prior to and during the peak cardiovascular responses: pre-peak/baseline (6 days before the peak cardiovascular response), and peak (6 days during the peak cardiovascular response).

## Results

### Case I

#### Demographic Characteristics and COVID-19 Symptoms and Experiences

Case I participated in the study from 11 to 24 weeks of gestation. She was 24 years old, self-identified as Hispanic, had completed some college-level education, was a single mother with two children, working part-time with a low-income. She was obese with a pre-pregnancy BMI of 33.2, was diagnosed with anemia during the current pregnancy, and had no other medical/pregnancy-related conditions. Case I reported experiencing mild COVID-19 symptoms including nausea, cough, loss of taste and smell, sore throat, and nasal congestion on December 25, 2020 (i.e., gestational week 16 day 0). She obtained a COVID-19 diagnosis on December 31, 2020 (i.e., gestational week 16 day 6). During a follow-up text message with her research assistant in January 2021, she shared that her COVID-19 symptoms had lasted about 2 weeks and was feeling better overall, but not fully recovered. Her text read, “*...It's going good...my nausea went away...well a little bit it's better now. I can eat, gained 7lbs, so happy… I got a little throat discomfort other than that I'm well*.” When asked whether she took a second test to confirm recovery, she stated, “*I haven't done a test and I believe so [I have recovered], just get out of breath at some point, I do not know if it's due to pregnancy*.”

Table 1 shows the physiological responses over the course of the COVID-19 infection for Case I. Compared to baseline, resting HR significantly increased and HRV decreased in the peak period. During the post-peak period, (i) resting HR and RR significantly dropped, while HRV increased, (ii) deep sleep significantly increased, whereas light sleep decreased.

**Table 1:** Case I: comparison of physiological responses to COVID-19 surrounding the period of infection.

**Variables**	**Pre-COVID-19 peak cardiovascular response, *n* = 4, mean (SD)**	**COVID-19 peak cardiovascular response, *n* = 3, mean (SD)**	**Post-COVID-19 peak cardiovascular response, *n* = 3 mean (SD)**	***T*-test (pre-peak vs. peak)**	***T*-test ** **(peak vs. post-peak)**	***T*-test (pre-peak vs. post-peak)**
^a^HR, beats per minute (bpm)	64.66 (2.57)	72.04 (2.65)	63.42 (0.65)	*p* = 0.014^**^	*p* = 0.005^***^	*p* = 0.461
^b^HRV (RMSSD), millisecond (ms)	55.00 (8.52)	40.33 (2.08)	46.33 (0.58)	*p* = 0.036^**^	*p* = 0.009^***^	*p* = 0.147
^c^RR, count	14.41 (0.37)	14.71 (0.38)	13.63 (0.66)	*p* = 0.342	*p* = 0.069^*^	*p* = 0.101
Skin temperature deviation, Celsius	0.05 (0.12)	0.16 (0.28)	−0.21 (0.12)	*p* = 0.506	*p* = 0.107	*p* = 0.036^**^
Sleep duration, hours	8.53 (0.98)	8.21 (1.42)	8.89 (1.49)	*p* = 0.730	*p* = 0.596	*p* = 0.716
Stages of sleep, hours						
Deep	2.09 (0.43)	1.77 (0.56)	2.73 (0.43)	*p* = 0.426	*p* = 0.078^*^	*p* = 0.110
^d^REM	1.82 (0.46)	1.65 (0.46)	2.17 (0.66)	*p* = 0.637	*p* = 0.326	*p* = 0.451
Light	3.90 (0.77)	4.03 (0.39)	3.12 (0.35)	*p* = 0.805	*p* = 0.038^**^	*p* = 0.167

* *p < 0.1,*

** *p < 0.05, and*

*** *p < 0.01*.

a *HR, resting heart rate*.

b *HRV, resting heart rate variability*.

c *RR, resting respiratory rate*.

d *REM, rapid eye movement*.

**Figure 1:**
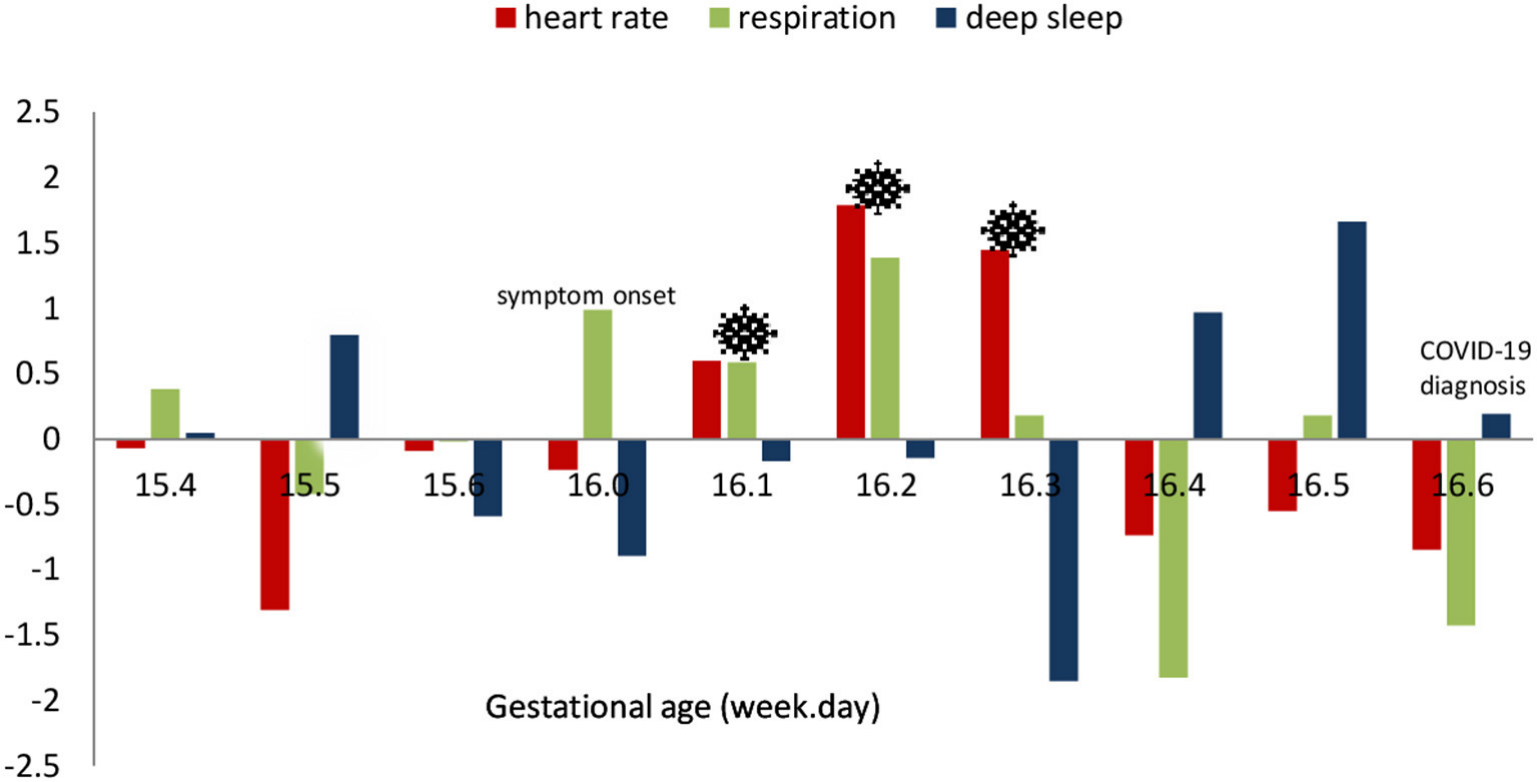
Case I: daily standardized scores of selected physiological parameters surrounding the period of COVID-19 infection.

Figure 1 demonstrates the standardized *Z*-scores of the daily changes for the three selected physiological indicators: resting HR, RR, and the deep sleep duration. We observed a significant increase in HR during the peak period (i.e., gestation week 16 days 1–3). This marked cardiovascular change occurred 1 day following the onset of symptoms (i.e., gestation week 16 day 0). Subsequently, resting HR and RR significantly decreased and deep sleep increased during the post-peak period (i.e., gestation week 16 days 4–6).

### Case II

#### Demographic Characteristics and COVID-19 Symptoms and Experiences

Case II participated in the study from 31 weeks of gestation until postpartum. She was 25 years old and self-identified as Hispanic, had a Bachelor's degree, was unemployed, and had a low family income. She was married, had one child, and lived with the father of the baby. She was overweight with a pre-pregnancy BMI of 27.5, and had no other medical/pregnancy-related conditions. Case II reported experiencing moderate COVID-19 symptoms on December 31, 2020 (i.e., gestation week 34 day 6), including fever, chills, sweating/flushing, headache, runny nose, nasal congestion, body/joint ache, fatigue, and loss of smell. She obtained a COVID-19 diagnosis on January 7, 2021 (i.e., gestation week 35 day 6). Case II said all her symptoms lasted about 2 weeks and did not require hospitalization. She also shared that her entire family contracted COVID-19 during that period. In late January 2021, she had a normal vaginal delivery at 38 weeks of gestation, giving birth to a healthy baby who tested negative for COVID-19. During the postpartum follow-up, she reported having no limitations in her daily life, no pain, depression, or anxiety. She also felt that her health had returned to normal without any post-COVID-19 symptoms.

Table 2 shows the physiological responses over the course of COVID-19 infection for Case II. During the 6-day peak period, resting HR and RR significantly increased, while HRV and deep sleep plummeted.

**Table 2:** Case II: comparison of physiological responses to COVID-19 surrounding the period of infection.

**Variables**	**Pre-COVID-19 peak cardiovascular response, *n* = 6, mean (SD)**	**COVID-19 peak cardiovascular response, *n* = 6, mean (SD)**	***T*-test (pre-peak vs. peak)**
^a^HR, bpm	71.46 (1.93)	78.56 (4.65)	*p* = 0.006^***^
^b^HRV, ms	58.33 (10.67)	41.42 (11.85)	*p* = 0.027^**^
^c^RR, count	13.98 (0.35)	14.47 (0.40)	*p* = 0.048^**^
Skin temperature deviation, Celsius	0.06 (0.16)	0.12 (0.30)	*p* = 0.661
Sleep duration, hours	9.42 (0.99)	8.05 (2.31)	*p* = 0.213
Stages of sleep, hours			
Deep	1.98 (0.33)	1.57 (0.22)	*p* = 0.029^**^
**^d^**REM	1.62 (0.36)	1.23 (0.59)	*p* = 0.198
Light	4.10 (0.23)	3.26 (1.32)	*p* = 0.157

** *p < 0.05, and*

*** *p < 0.01*.

a *HR, resting heart rate*.

b *HRV, resting heart rate variability*.

c *RR, resting respiratory rate*.

d *REM, rapid eye movement*.

**Figure 2:**
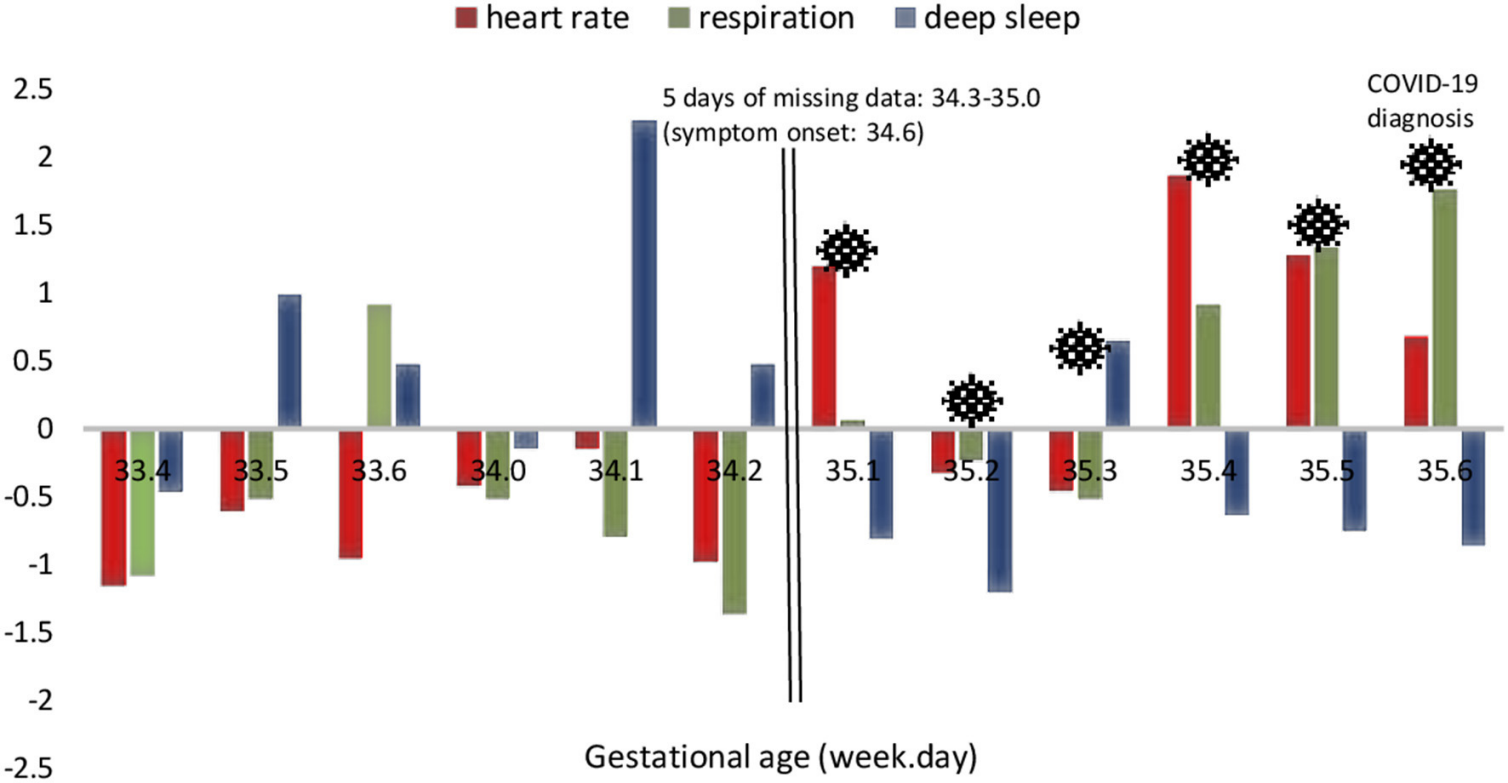
Case II: daily standardized scores of selected physiological parameters surrounding the period of COVID-19 infection.

Figure 2 is a visual representation of the daily changes in resting HR, RR, and deep sleep using the standardized Z-scores. Resting HR and RR increased significantly from baseline (i.e., gestation weeks 33–34) to the peak period (i.e., gestation week 35 days 1–6), whereas deep sleep decreased. Case II was unable to wear the *Oura* ring for 5 days surrounding the onset of symptoms (i.e., gestation week 34 day 6) due to her illness and that of her family. Therefore, we could not determine the exact days that resting HR, RR, and deep sleep changed prior to or following symptom onset.

## Discussion

To the best of our knowledge, this is one of the first studies to continuously monitor the physiological changes in pregnant women daily using a wearable device over the course of COVID-19 infection. During the peak physiological responses to COVID-19, our results showed a significant elevation in resting HR, RR and a reduction in HRV. These changes were evident in Case I one day following the onset of symptoms. In two recent studies that examined physiological signs using a wearable device in non-pregnant subjects diagnosed with COVID-19, similar cardiorespiratory patterns (elevated resting HR and RR, and decreased HRV) were found surrounding the onset of symptoms (17, 19). Our findings support both studies and suggest that cardiorespiratory responses may occur as early as the start of symptoms. Notably, our study provides initial evidence on how pregnant women's cardiorespiratory system adapts to mild-to-moderate COVID-19 illness.

We also found significant changes in the stages of sleep (i.e., deep sleep), but not in sleep duration during the peak physiological response to COVID-19. Our results differed from those of two recent studies that found an increase in sleep duration in non-pregnant individuals infected with COVID-19, using various wearable devices such as Fitbits, Apple Watches, and other devices (16, 18). Growing literature shows that altered sleep responses to infections (e.g., longer sleep/sleepiness during illness) indicates immunity adaptation in terms of increasing body temperature (fever) that activates white blood cells, inhibits viral growth, and promotes recovery (27, 28). The discrepancy of sleep duration between our study and prior studies (16, 18) may be related to a few factors such as COVID-19 symptom manifestations (e.g., extent of temperature change), COVID-19 severity, selected observation time or wearable devices. Importantly, pregnant women may have different physiological responses to COVID-19 particularly for sleep duration and stages due to the expected cardiorespiratory and sleep patterns exhibited during pregnancy itself, together with the added effects of COVID-19. Our study provides preliminary evidence on the different sleep patterns potentially altered by COVID-19, emphasizing that sleep stages (deep sleep in particular) may provide promising metrics to track COVID-19 progression during pregnancy.

There have been varied reports regarding the time course of the COVID-19 infection among non-pregnant individuals (19, 29, 30). Our results extended the existing literature by including the observed progression of COVID-19 in pregnant women with mild-to-moderate symptoms. We found that significant changes in peak physiological responses lasted for 3–6 days. Additionally, self-reported COVID-19 symptoms by both cases indicated that symptoms lasted about 2 weeks. The physiological and self-reported clinical manifestations in our pregnant women appeared to fall within the range of the estimated time course for mild and moderate symptoms with non-pregnant individuals (i.e., 11–16 days) (19). Our findings suggest the potential to use the changes in cardiorespiratory responses and sleep for real-time monitoring of the progression of infection and health in pregnant women.

The effects of COVID-19 on the fetus are still largely unknown. Case II had a normal vaginal delivery at 38 weeks of gestation, with no clinical evidence of vertical transmission. Notably, in a systematic review of 19 studies with 70 women, pregnant women hospitalized with COVID-19 were likely to experience preterm birth (i.e., <37 weeks of gestation), caesarian delivery and preeclampsia, but no vertical transmission (31).

### Strengths and Limitations

The major strength of this study lies in the availability of continuous data which allowed a micro-level examination of the cardiovascular, respiratory, and sleep changes that occurred during the period surrounding COVID-19 infection. Pregnancy is characterized by variations in physiology. Our study provided a snapshot of these physiological changes that occur during pregnancy and the specific responses to COVID-19 infection. Recruitment of underserved low-income women in the parent study provided the opportunity to describe COVID-19 physiological responses in this sample. In terms of limitations, due to the inability of Case II to wear the smart ring during the period of declined health, data were not available for 3 days prior to and 2 days immediately following the onset of symptoms. However, we were able to compare pre-and post-COVID-19 physiological changes using the data available on other dates (Figure 2). Another limitation was that both cases had a pre-pregnancy BMI above the normal range which may have influenced the observed effects. In addition, data on sleep disorders was not collected in our study. Some studies found that pregnant women with sleep apnea had reduced olfactory function compared to non-pregnant women (32, 33). Furthermore, only Case II reported her birth outcome. Lastly, the case study design limits the interpretability and generalizability of results.

## Conclusion

This study suggests that resting HR, HRV, RR, and sleep stages are important and sensitive indicators of COVID-19 infection during pregnancy. The continuous monitoring of such measures is vital to track health and well-being, and thus, provides appropriate and safe clinical care or triage for pregnant women. There is a need for longitudinal studies to further investigate the impact of infections, such as COVID-19, on the health and well-being of pregnant women and their infants.

## Data Availability Statement

The raw data supporting the conclusions of this article will be made available by the authors, without undue reservation.

## Ethics Statement

The studies involving human participants were reviewed and approved by University of California, Irvine Institutional Review Board. Written informed consent for participation was not required for this study in accordance with the national legislation and the institutional requirements.

## Author Contributions

TJ, PK, HB, PP, AR, ND, and YG contributed to the design and supervision of the study. TJ, PK, and YG conceived the manuscript. TJ and YG reviewed the literature. TJ, PK, HB, and YG oversaw the data collection. TJ conducted the analysis and wrote the initial manuscript. YG and PK revised and finalized the manuscript. All authors reviewed and approved the final manuscript.

## Funding

This research was funded by the US National Science Foundation under the Smart and Connected Communities (S&CC) Grant CNS-1831918.

## Conflict of Interest

The authors declare that the research was conducted in the absence of any commercial or financial relationships that could be construed as a potential conflict of interest.

## Publisher's Note

All claims expressed in this article are solely those of the authors and do not necessarily represent those of their affiliated organizations, or those of the publisher, the editors and the reviewers. Any product that may be evaluated in this article, or claim that may be made by its manufacturer, is not guaranteed or endorsed by the publisher.
